# Progress in Molecular Mechanisms of Depression Induced by Mother-Infant Separation and Intervention of Traditional Chinese Medicine

**DOI:** 10.31083/AP45684

**Published:** 2025-08-11

**Authors:** Ling-hui Kong, Min Liu, Hui Li, Rui-rui Shang, Shi-Meng Lv, Zhong-lin Wang, Qiang Ren

**Affiliations:** ^1^The First Clinical Medical College of Shandong University of Traditional Chinese Medicine, 250014 Jinan, Shandong, China; ^2^Emergency Department, Second Affiliated Hospital of Shandong University of Traditional Chinese Medicine, 250014 Jinan, Shandong, China; ^3^Affiliated Hospital of Shandong University of Traditional Chinese Medicine, 250014 Jinan, Shandong, China; ^4^College of Rehabilitation Medicine, Shandong University of Traditional Chinese Medicine, 250355 Jinan, Shandong, China; ^5^Department of Neurology, Second Affiliated Hospital of Shandong University of Traditional Chinese Medicine, 250014 Jinan, Shandong, China

**Keywords:** depression, maternal separation, early life stress, molecular mechanisms, traditional Chinese medicine, research progress

## Abstract

Depression is a serious mental and emotional disorder and is considered to be the greatest cause of non-fatal disease worldwide. Early life stress (ELS) refers to the exposure of an individual to physical and psychological stress events such as neglect or abuse in early life that has a long-term impact on brain development, thus inducing emotional and cognitive disorders in adulthood. It is the main susceptibility and risk factor for depression. Current clinical treatment is primarily based on Western medicines such as fluoxetine, but there can be serious adverse reactions. Therefore, exploring the biochemical mechanism of ELS-induced disorders and how to intervene effectively and safely to prevent and treat depression has become a significant issue. Traditional Chinese medicine (TCM) has the unique advantages of few adverse reactions and high safety and has great potential for the treatment of depression. Maternal separation (MS) is one of the most important and commonly used models for simulating ELS. Many studies have shown that MS-induced depression involves the regulation of multiple pathways and several studies have shown that TCM improves MS-induced depression. However, there is currently a lack of systematic reviews and summaries of the molecular mechanisms of MS-induced depression and traditional Chinese medical interventions. Therefore, the mechanisms of action and traditional Chinese medical interventions for MS-mediated depression were reviewed by searching recent relevant literature and discussing the limitations of current research. The aim was to provide guidance for follow-up basic research and clinical treatment.

## Main Points

1. MS serves as a core animal model for early-life stress-induced depression.

2. MS mediates depression through eight molecular pathways including neuroinflammation, neural plasticity, and HPA axis dysregulation.

3. TCM monomers (e.g., resveratrol, Ganoderma triterpenes) and compounds (e.g., Sini San) ameliorate depressive behaviors via multi-target mechanisms.

4. TCM treats depression by regulating the gut microbiota-brain axis, epigenetic modifications, and autophagy.

5. Current research requires expansion to address TCM quality control and clinical translation bottlenecks.

## 1. Introduction

Depression is a serious mental and emotional disorder. It is considered to be the leading cause of non-fatal disease worldwide and it is predicted that by 2030 depression will be the leading global disease burden [1, 2]. Early life stress (ELS) refers to the impact of stressful physical and psychological events such as neglect or abuse (adverse stimuli such as mother-child separation, social failure, and negative family relationships) on an individual at an early stage of life [3]. Such events have long-term effects on brain development, thereby inducing emotional and cognitive disorders in adulthood. It is the main susceptibility and risk factor for depression [4, 5, 6]. However, current clinical protocols predominantly rely on selective serotonin reuptake inhibitors (SSRIs), which are nevertheless associated with significant adverse effects including gastrointestinal distress, cephalalgia, persistent sexual dysfunction, and metabolic dysregulation. Moreover, these therapeutic agents frequently exhibit delayed therapeutic onset and substantial non-response rates [7, 8]. Exploring the biochemical mechanism of ELS caused depression and how to intervene effectively and safely to prevent and treat it has become a significant issue that needs to be addressed.

Traditional Chinese medicine (TCM) operates through multicomponent, multitarget, and multipathway mechanisms that synergistically achieve systemic modulation. Pharmacologically active TCM constituents exhibit clinically validated antidepressant effects with favorable safety profiles, demonstrating substantial potential for novel antidepressant development [9]. One of the most important and commonly used models for imitating adverse early experiences in human childhood is maternal separation (MS) [10]. Patients with depression induced by MS are accompanied by clinical characteristics such as anhedonia, attachment disorder, and social withdrawal [11]. Accumulating evidence demonstrates that MS modulates depression pathogenesis through multifaceted pathways, while TCM exhibits critical therapeutic effects in MS-associated depression models. Nevertheless, a comprehensive mechanistic elucidation of both MS-induced depression and corresponding TCM interventions remains absent from the literature. This review synthesizes recent advances by systematically analyzing the pathological networks underlying MS-related depression and current phytotherapeutic modulation strategies, aiming to establish evidence-based insights for future translational research and clinical practice.

## 2. Molecular Mechanism of Depression Mediated by Maternal Separation

An animal maternal and infant separation stress model is widely used with rats and mice. In MS, female mice are separated (1–24 hours) from their offspring. In the MS animal model, female mice provide key survival resources such as nutrition and sensory stimulation for their young. Short-term separation simulates the natural nest-leaving behavior of female mice, while long-term mother-infant separation causes severe environmental deprivation, which induces weakened anterior pulse inhibition, separation anxiety, depressive-like behaviors, and cognitive impairment in young mice [12, 13].

### 2.1 Neuroinflammation

Substantial empirical evidence has established a robust correlation between depressive disorders and neuroinflammatory pathways. Proinflammatory activation not only predisposes individuals to depressive disorders but also exacerbates disease progression, with elevated proinflammatory mediators and administration of exogenous proinflammatory agents significantly amplifying depression risk in clinical populations. When antidepressants are used, the peripheral inflammatory cytokine levels of depressed patients decrease [14]. Collapsin response mediator protein (CRMP) is a widely expressed phosphoprotein that coordinates cytoskeleton formation and regulates cell division, migration, polarity, and synaptic connections. Collapsin response mediator protein 2 (CRMP2) is one of the more studied molecules and has an important role in the nervous system [15]. Current research confirms that experiencing short-term MS has a potentially protective effect on the nervous system, but long-term MS activates neuroinflammation and destroys neuroprotection [16].

Microglia are cells of mesodermal origin in nervous tissue. Activated microglia are a major source of pro-inflammatory cytokines and inflammation-related proteins regulated by various intracellular signals [17]. Recent reports have shown that mice exposed to MS stress early in life have increased long-term mood changes (e.g., depressive-like behavior) in adolescence and adulthood, with a more pronounced response in female mice. Abnormal behavior is associated with neuroinflammation caused by activated microglia and a tryptophan-kynurenine metabolic disorder [18]. Interleukin (IL)-17 was the first member identified in a new family of proinflammatory cytokines [19]. Evidence supports that exposure to cumulative mild stress promotes long-term depressive symptoms in mice through upregulation of IL-17 and it is believed that IL-17 may be an important potential target for antidepressants [20].

Jumonji domain-containing protein 3 (JMJD3) is a key enzyme in histone methylation modification. By specifically removing the trimethylation modification of histone H3 at lysine 27 (H3K27me3), JMJD3 potentiates neuroinflammatory cascades in rat prefrontal cortical and hippocampal microenvironments, mechanistically driving susceptibility to metabolic syndrome-associated depressive phenotypes [21, 22].

The phosphatidylinositol 3-kinase (PI3K)/protein kinase B (AKT) pathway is one of the core signal pathways in cells that regulates cell growth, proliferation, movement, metabolism, and survival [23]. It also activates its downstream NF-κB to induce neuroinflammation [24]. Current reports indicate that neonatal maternal deprivation combined with chronic mild stress more effectively establishes a depression model in adolescent female rats. This model may be closely related to the activation of microglia and upregulation of the PI3K/AKT/NF-κB signaling pathway [25].

Regulating neuroinflammation improves depressive-like behavior caused by MS. Enrichment increases the complexity and novelty of the physical and social environment and has been shown to have multiple benefits for the body [26]. It improves neuroinflammation, neuronal apoptosis, synaptic plasticity damage, and depressive-like behavior in female rats experiencing postpartum depression induced by MS [27]. Progesterone is a hormone based on progesterone and its analogues can act as local neurosteroids [28]. Its administration significantly alleviates MS-induced depressive-like behavior and improves the neuroimmune response and excessive oxidative stress in mouse hippocampus [29].

### 2.2 Neural Plasticity

#### 2.2.1 Structural Plasticity

Neuroplasticity exhibits two main types, either structural or functional. Structural plasticity involves promoting neurogenesis, dendritic spine formation, and changes in axon growth and repair mechanisms, including changes in the number and connection of synapses, the density of dendritic spines, elongation or shrinkage of nerve endings (axons and dendrites), and even changes in the number of neuronal cells [30, 31]. ELS-induced depressive-like models seriously affect the development of the mouse brain [32]. Adult hippocampal neurogenesis refers to the complete process of proliferation and division of hippocampal neural stem cells into neural progenitor cells, gradual migration to functional areas, continuous plastic changes, and establishment of synaptic connections with other neurons [33], which play an important role in structural plasticity. However, long-term MS leads to depressive states by damaging postnatal dentate gyrus neurogenesis [34]. Animals exposed to MS also show early-onset age-related depression and altered metabolic risk, that are effects associated with altered hippocampal neurogenesis [35]. Brain-derived neurotrophic factor (BDNF) is a widely studied growth factor that has an important role in mediating processes such as neuronal maturation, synapse formation, and synaptic plasticity in the brain [36]. A recent study found that prolonged MS (PMS, 180 minutes of separation per day) suppresses BDNF expression in the prefrontal cortex (PFC) by elevating cortisol (CORT) levels [37]. MS also reduces BDNF protein and mRNA levels when inducing a depressive-like phenotype [38]. The AKT/glycogen synthase kinase-3β (GSK3β)/CRMP2 pathway plays a role in neural development [39], while early maternal deprivation alters the cytoskeleton and induces depressive-like behavior in adult male rats [40] by impairing the normal expression and activity of the AKT/GSK3β/CRMP2 signaling pathway.

#### 2.2.2 Synaptic Plasticity

Unlike structural plasticity, functional plasticity adjusts synaptic changes between neurons without changing the structure, such as by long-term potentiation (LTP) and long-term depression (LTD) [41, 42]. LTP and LTD are two mechanisms that affect the impaired cognitive and affective functions of Major Depressive Disorder (MDD) patients. Under strong and continuous stimulation, neuronal discharge increases, followed by an increase in LTP by enhancing synapses that mediate learning and memory. On the other hand, LTD is a decrease in the efficacy of synapses and activity-dependent reduction in the connectivity of neurons [43].

Huang *et al*. [44] found that adult female rats that experienced MS and chronic unpredictable mild stress (CUMS) exhibited more severe depressive-like behavior and had fewer Nissl bodies in the hippocampal cornu ammonis 1 (CA1) and dentate gyrus (DG) regions and the expression of synaptophysin, postsynaptic density-95, and growth-associated protein-43 was downregulated. MicroRNAs are small endogenous RNAs that regulate gene expression post-transcriptionally [45]. It has been previously reported that miR-34c may be involved in the pathogenesis of depression by regulating neuroplasticity, stress response, and other biological processes [46]. Importantly, the miR-34c-5p synaptophysin 1 pathway is involved in the susceptibility to MS-induced depression by regulating neuroplasticity in the mouse hippocampus [47].

Conversely, animals with different stress vulnerabilities were grouped using an MS model and synaptic responses in the lateral habenula were studied. The results showed that LTD was impaired in the susceptible group and extrasynaptic LTD was enhanced [48]. Neurons in the basolateral amygdala play an important role in depression [49]. Dysregulation of neuronal activity and synaptic transmission in projection neurons in that region plays an important role in the pathological behavior of mice induced by MS [50]. Cui *et al*. [51] used metabolomics to show that the MS-induced rat depression model involves damage to synaptic plasticity and metabolic disturbances. Alternatively, when MS is combined with chronic restraint stress, it also inhibits the hippocampal mechanistic target of rapamycin (mTOR) pathway, thereby reducing synaptic plasticity [52]. In summary, neuroplasticity mediates MS-induced depressive-like behavior and regulation based on neuroplasticity is a potentially effective therapeutic target.

### 2.3 Hypothalamic-Pituitary-Adrenal Axis

The hypothalamic-pituitary-adrenal (HPA) axis is an important part of the neuroendocrine system that controls the stress response. When the HPA axis is activated, the paraventricular nucleus of the hypothalamus releases corticotropin-releasing hormone (CRH), which signals the anterior pituitary gland to secrete adrenocorticotropic hormone (ACTH) into the bloodstream. ACTH acts on the adrenal cortex, stimulating the secretion of CORT [53]. Hyperfunction of the HPA axis is an important factor in the pathogenesis of depression. Increased CRH, ACTH, and glucocorticoids, a disorder of negative feedback in the HPA axis, enlargement of the pituitary gland and adrenal glands, and hypercortisolism have been found in some depressed patients [54].

Early life stress alters acute corticosterone-induced synaptic plasticity in the medial prefrontal cortex of adolescent rats [55]. Animals exposed to ELS exhibit a long-term increase in hypothalamic *CRH* mRNA levels and a reduced plasma corticosterone response [56], and MS exacerbates HPA axis hyperactivity and endocrine pancreatic dysfunction under chronic social defeat stress [57]. Alternatively, hyperactivity of the HPA axis may induce a detrimental effect of MS on behavior following MS, changes in microbiota composition, and activation of neuroimmune responses [58]. Interestingly, higher hair CORT concentrations have been found in clinical settings in individuals whose mothers divorced during childhood. This effect is independent of a variety of factors, suggesting a lifelong pathway between early life separation and HPA function in old age [59].

### 2.4 Neurotransmitters

Monoamine neurotransmitters are central nervous system neurotransmitters that mainly consist of two categories: catecholamines and indolamines. Catecholamines include dopamine (DA), norepinephrine (NE), and epinephrine, while indolamines mainly include 5-hydroxytryptamine (5-HT). DA is an important regulator of learning and motivation [60], while 5-HT and NE are primarily involved in regulating emotional cognition and sleep. When there is a disorder of monoamine neurotransmitters, it leads to various emotional changes [54]. It has been shown that the main cause of the onset of depression is not the secretion of neurotransmitters and that drugs that increase the synaptic concentration of monoamines improve the symptoms of depression [61].

A recent study has shown that MS causes long-term disturbances of the serotonergic system and lead to anxiety and depressive-like behavior [62]. Lipopolysaccharide (LPS), a component of the outer cell wall of Gram-negative bacteria, is a substance composed of lipids and polysaccharides. The LPS-induced depressive-like model is often used to study the mechanism of inflammation-related depression and the therapeutic effect of drugs [63]. Yu *et al*. [64] compared LPS with MS as a method of inducing depression and found that although LPS induced stronger systemic inflammation, importantly, MS impaired the function of the HPA axis and 5-HT system (significant reduction in 5-HT levels in the hippocampus and PFC). However, in the MS-induced depression model, the improvement in depressive-like behavior involves the modulation of neurotransmitters. Zolfaghari *et al*. [65] found that adolescent treatment with wheel running and fluoxetine reduced MS-induced depressive-like and anxiety-like disorders in adult male rats and these effects were accompanied by a normalization of serum CORT and gene expression related to serotonin signaling in the hippocampus and PFC.

### 2.5 Microbiota-Gut-Brain Axis

The intestinal microbiota has recently been recognized as a major internal metabolic organ, consisting of >10^14^ microorganisms with a total mass of approximately 0.3% of the body mass of an individual [66]. When intestinal flora is disturbed it damages the immune and central nervous systems through flora-gut interactions and gut-brain communication, inducing the pathological development of depression [67].

MS effects intestinal microorganisms. In captive giant pandas it was found that early MS may affect the stress caused by an adverse early rearing environment, which is related to the intestinal microbiota of captive adult giant pandas [68]. Importantly, MS induces peripheral and central inflammation and tryptophan (TRP)-kynurenine (KYN) pathway metabolism in a sex-dependent manner, as well as sex-specific changes in intestinal microorganisms that potentially induce depressive-like phenotypes [69]. Alternatively, *Bacillus coagulans* Unique IS-2 mediates its antidepressant effect by remodeling the gut-brain axis of the microbiome in a rat model of MS combined with CUMS [70]. The gut microbiota and its metabolites mediate the therapeutic effect of a probiotic mixture on MS-induced brain dysfunction [71]. A multi-strain probiotic and glutamine formulation (Cogniol) improved the depressive-like phenotype induced by MS combined with CUMS by reshaping the gut microbiota-brain activity in both sexes [72]. *Lactobacillus casei*, one of the most commonly used probiotics for the treatment of gastrointestinal-related diseases, has potential therapeutic effects on depression [73]. A recent study has shown that *L. casei* treats postpartum depression by regulating the brain-derived neurotrophic factor (BDNF)-extracellular signal-regulated kinase 1/2 (ERK1/2) pathway, altering the composition of the intestinal flora, brain monoamines, and oxidative stress [74]. *B. pseudocatenulatum* CECT 7765 beneficially modulates the early-life overactivation of the HPA axis caused by MS by regulating the intestinal neurotransmitter and cytokine network, which has both short- and long-term effects on brain biochemistry and behavior, with long-term effects extending into adulthood [75].

### 2.6 Epigenetics

Epigenetics include stable changes in gene expression controlled by transcriptional, post-transcriptional, translational, or post-translational processes, including DNA modification, chromatin remodeling, histone modification, RNA modification, and non-coding RNA regulation, without any changes to the DNA sequence. The risk of MDD is affected by a combination of genetic and environmental factors and the interaction between genes and the environment is determined by epigenetic mechanisms, which may be a major pathogenic factor in depression [76]. Importantly, epigenetic mechanisms play a significant role in antidepressant research. It has been reported that methylation-specific oxytocin receptor genes in the hippocampus may play an important role in the susceptibility to depression induced by early life stress and that the 5-HT/NE/DA triple reuptake inhibitor LPM570065 may reduce depression susceptibility by reversing methylation of the oxytocin receptor gene [77]. MS also enhances epigenetic regulation of the *BDNF* gene in response to stress in infancy and subsequently in adulthood, potentially increasing susceptibility to stress [78]. Additionally, MS induces epigenetic changes in the BDNF exon I promoter, changes that are blocked during adulthood by antidepressant treatment [79]. It has also been reported that MS has a long-term negative effect on behavior by modifying histones on the glucocorticoid receptor gene throughout the life cycle [80].

### 2.7 Autophagy

Autophagy is a process in which cells degrade and recycle proteins and organelles to maintain homeostasis. It plays a protective role in cells, but disruption of the autophagy mechanism or excessive autophagy flux usually leads to cell death [81]. Dysregulation of autophagy is closely related to the development of depression pathology. Previous studies have found that in a CORT-induced depression model, neurons are hyperactive in autophagy and deplete BDNF, inhibiting adult hippocampal neurogenesis [82], with the autophagy process being related to the activation of nod-, lrr-, and pyrin domain-containing protein 3 (NLRP3) inflammasomes. Dysfunctional lysosomes in the autophagy-lysosome pathway may disrupt the degradation of NLRP3 inflammasomes and promote the production of pro-inflammatory factors, leading to depressive-like behavior in mice [83]. However, regulation of autophagy improves depressive-like behavior [84, 85].

MS also effects autophagy. Recent reports have shown that MS induces different autophagy responses in the hippocampus and PFC (autophagy is inhibited in the hippocampus, while activated in the PFC), and is potentially affected by the N-methyl-D-aspartate receptor subunit 2B (NR2B) signaling pathway [86]. Further research has also found that MS causes brain dysfunction in adult rats, which involves the regulation of hippocampal neuronal autophagy through leucine metabolism in the cerebrospinal fluid [87].

### 2.8 Circadian Rhythm

Circadian rhythmicity is generated within a genetically encoded molecular clock, where the components interact to produce periodic changes in their abundance and activity with a period of approximately 1 day [88]. The importance of time has always been prevalent in the human world and disruptions to normal light/dark and sleep/wake cycles are now the norm rather than the exception for a large proportion of the population, while MDD is strongly associated with abnormal sleep and circadian rhythm in various physiological processes. Disruptions to normal sleep/wake patterns, light/dark changes and seasonal changes in the environment may trigger depressive episodes [89], while regulation based on disturbed circadian rhythms improves depressive-like symptoms [90].

**Fig. 1.**
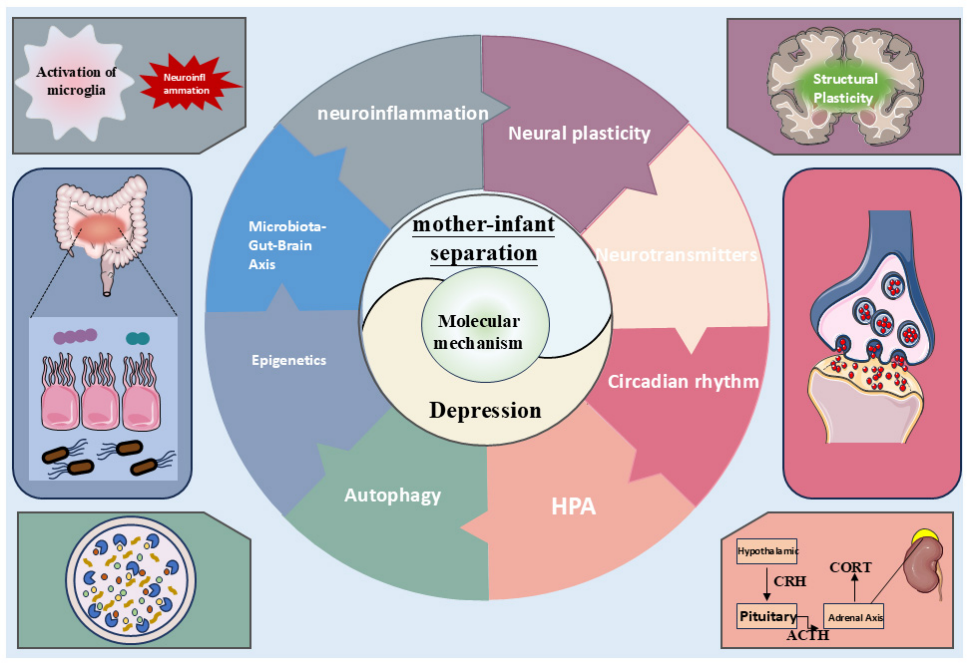
**Schematic diagram of the molecular mechanism of depression induced by maternal separation**. HPA, hypothalamic-pituitary-adrenal; CRH, corticotropin-releasing hormone; ACTH, adrenocorticotropic hormone; CORT, cortisol. Figure created using office software (office 2024, Microsoft Corporation, Redmond, WA, USA).

It has been reported that MS is associated with altered circadian patterns of CORT in midlife [91] and that animals exposed to MS have higher core body temperatures during the dark phase of the circadian cycle. MS causes changes in the body’s thermoregulatory pattern that persist into adulthood [92]. The above-mentioned changes in the body’s biological clock rhythm system caused by MS are directly or indirectly involved in the pathogenesis of MDD (Fig. 1).

## 3. Mechanism of Action of TCM

### 3.1 Active Ingredients of TCM

Resveratrol is a nutrient with multiple benefits. It is a natural phytoalexin produced by plants to protect them from environmental stress and pathogen invasion [93]. Resveratrol has anti-inflammatory and anti-oxidative stress effects and it has therapeutic effects on central nervous system diseases such as major depression, bipolar disorder, Alzheimer’s disease, and autism [94]. Previous studies have found that resveratrol exerts antidepressant effects by regulating autophagy and inhibiting neuroinflammation [95, 96]. Sirtuin 1 (SIRT1) is considered to be a metabolic sensor because it couples the metabolic state of cells to chromatin structure. The SIRT1/NF-κB signaling pathway is involved in the regulation of inflammatory responses [97]. In an animal model of MS-induced depression, MS caused animals to exhibit depressive-like behavior and elevated levels of pro-inflammatory cytokines and the SIRT1/NF-κB signaling pathway was dysregulated. Treatment with resveratrol improved these pathological phenomena [98]. On the other hand, resveratrol also improves the levels of brain antioxidants and monoamines, as well as the neuroprotective effect of HPA axis dysfunction and treats the depressive-like behavior induced by MS in rats [99].

The main alkaloid component of fenugreek, trigonelline, has been shown to have a variety of biological activities, including anti-diabetic and anti-cancer effects [100]. It has previously been reported that fenugreek seed extract alleviates LPS-induced learning and memory impairment in rats [101]. Research has also found that fenugreek seed extract is a promising drug for the treatment of various neurological diseases through network pharmacology and molecular docking [102]. In a model of MS, Lorigooini *et al*. [103] found that fenugreek seed extract exerts its antidepressant effect by alleviating oxidative stress and increasing antioxidant capacity.

*Ganoderma lucidum* has been used for centuries in Asian countries as a traditional medicine for the prevention and treatment of various diseases [104] and ganoderic triterpenoids (GLTs) are one of the main active ingredients in Ganoderma, which have various pharmacological effects such as anti-cancer [105]. Mi *et al*. [106] found that MS increased anxiety and depressive-like behavior in male and female mice, but subchronic administration of GLTs (40 mg/kg) in adulthood improved these pathological behaviors. Further mechanistic studies found that GLTs inhibited the expression of pro-inflammatory cytokines and the activation of microglia.

Oleanolic acid (OA) is a pentacyclic triterpenoid compound that is widely found in the plant kingdom and has received significant attention from the scientific community due to its biological activity against a wide range of diseases [107]. Ursolic acid (UA) is a natural pentacyclic triterpenoid compound extracted from a variety of traditional medicinal plants and most fruits and vegetables and has a wide range of therapeutic potential [108]. In a study combining OA and UA, it was found that OA was more effective than UA in reversing MS-induced depressive-like behavior and that its anti-depressant mechanism involved relieving neuroinflammation and improving synaptic plasticity [109].

Paeoniflorin is one of the active ingredients derived from *Paeonia albiflora*, which has a variety of pharmacological effects [110]. Paeoniflorin significantly improves the depressive-like behavior of MS mice. Its mechanism of action involves inhibiting the activation of microglial cell function [111]. Soy isoflavones are mainly found in legumes and are an important secondary metabolite with strong biological activity synthesized via the phenylpropanoid pathway [112]. A study found that soy isoflavones improve the depressive-like behavior of female rats experiencing MS and that higher concentrations of soy isoflavones (30 mg/kg⋅d^-1^) are more effective. A mechanistic study found that they increase serum dopamine and estrogen receptor β levels [113].

### 3.2 TCM Compounds

Practitioners of TCM believe that depressive disorders fall under the category of ‘depression’ and are a type of disease that TCM is good at treating and preventing. Patients with liver *qi* stagnation and an unsettled spirit are considered to suffer from a mood disorder. Among these disorders, the key is the liver’s failure to regulate *qi*, so treatment focuses on ‘liver regulation and depression relief’. The classic prescription Si Ni San was first recorded in Shang Han Lun. This prescription is composed of herbs such as *Bupleurum* and *Radix Paeoniae Alba* and is currently the basis of antidepressant treatment in TCM [114]. It has been reported that in the ELS model, Sini San exerts its antidepressant effect by regulating Rac1 activity and dendritic spine plasticity in the nucleus accumbens [115]. Additionally, it has been found that Sini San treatment of MS-induced depressive-like behavior involves the regulation of the BDNF/protein kinase A (PKA)/cAMP response element-binding protein (CREB) pathway [116]. It also activates the calcium-sensitive receptor (CaSR)-protein kinase C (PKC)-ERK signaling pathway to improving neuroplasticity [117] and also has a regulatory effect on mitochondrial dysfunction [118].

Pan and Yue [119] and others believe that depression has the pathogenesis of yang deficiency and poor *qi* circulation, which is closely related to neuropsychological changes caused by early trauma. Treatment needs to warm and supplement yang and promote *qi* to relieve depression. They formulated the Wenyang Jieyu Fang (formed by combining Erxian Tang and Xiaoyao San, which relieves depression). Pharmacological studies have found that the Wenyang Jieyu formula inhibits central pain hypersensitivity and regulates the function of the HPA axis by enhancing the expression of glucocorticoid receptors in the amygdala and inhibiting neuroplasticity and excitability in the amygdala region, thereby relieving depressive behavior and improving somatic pain hypersensitivity [120]. A comprehensive therapy that includes the Wenyang Jieyu formula improves the depressive model of MS combined with restraint stress or MS combined with LPS. The mechanism of action involves regulating the HPA axis and neuroplasticity, while inhibiting the activation of hippocampal microglia, thus relieving hyperactive neuroinflammation [121, 122].

Randomized, double-blind, placebo-controlled experiments conducted with clinical patients better reflect the true impact of TCM compound prescriptions on depression. A study showed that the TCM compound Kaixin San (composed of *Ginseng radix*, *Acori tatarinowii ahizoma*, *Poria, Polygalae Radix*) significantly improves the depressive symptoms and cognitive function of patients with mild-to-moderate depression.

Moreover, it reduces the ratios of low-density lipoprotein/high-density lipoprotein (LDL/HDL) and apolipoprotein B/apolipoprotein A1 (ApoB/ApoA1), and the level of apolipoprotein C3 (ApoC3) in the serum of patients with hyperlipidemia.

**Fig. 2.**
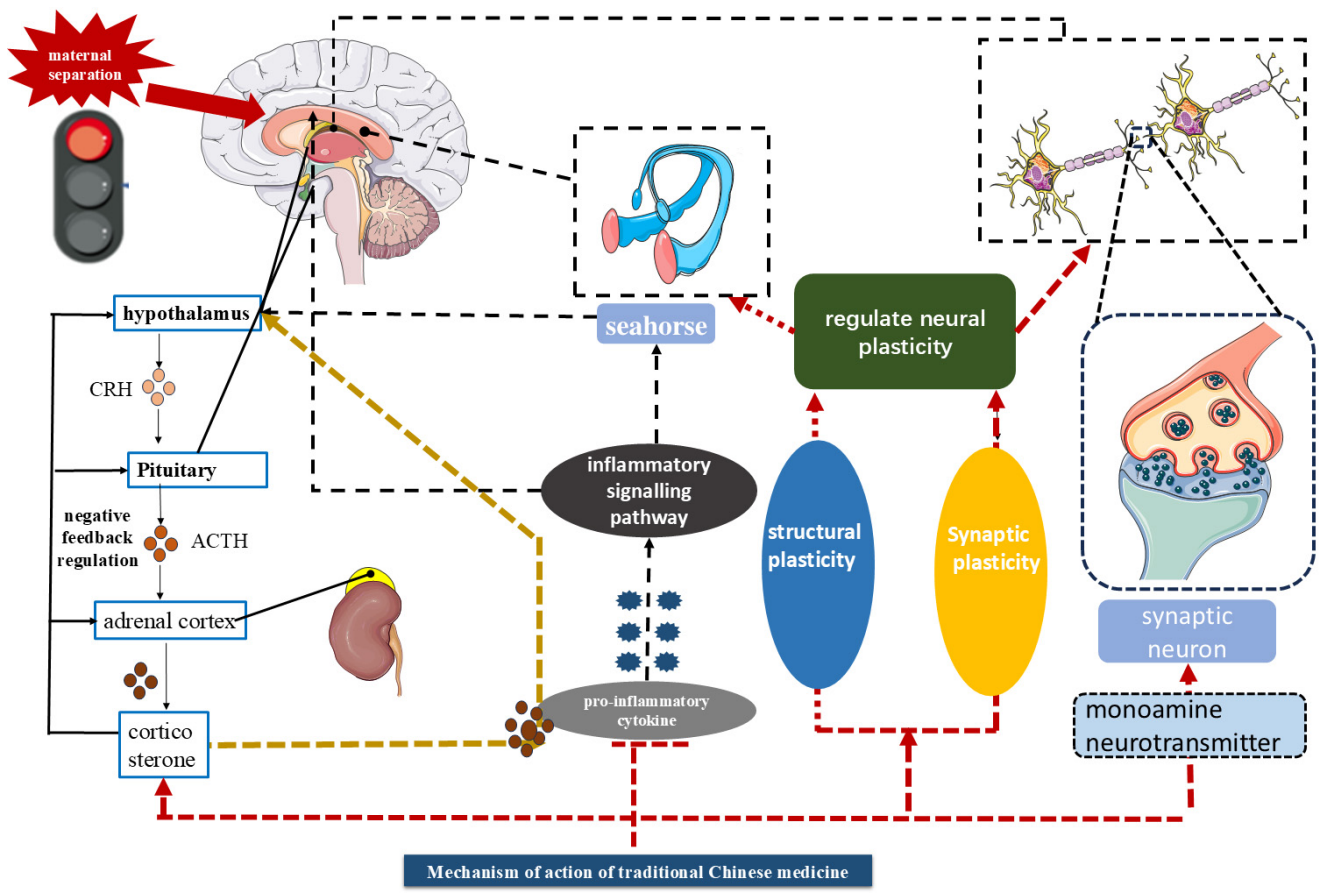
**Mechanism of action of traditional Chinese medicine**. Figure created using Office software.

It is indicated that this formula is applicable to patients with depression who have abnormal lipid metabolism and cardiometabolic diseases [123]. Proteomic analysis showed that Kaixin San stimulates the differential expression of proteins in a rat model of chronic mild stress and that these proteins could be involved in neural development and regeneration as well as synaptic remodeling [124]. Shenzhiling (SZL) is a tablet composed of Kaixin San. The outcome of a randomized, placebo-controlled, double-blind study on the effect of SZL on patients with mild-to-moderate depression compared with fluoxetine showed that the efficacy and safety of SZL were comparable to those of fluoxetine [125] (Fig. 2, Table 1, Ref. [98, 99, 103, 106, 109, 111, 113, 115, 116, 117, 118, 122, 123, 125]).

**Table 1.** **Mechanism of action of traditional Chinese medicine**.

Chinese medicine active ingredient/Chinese medicine compound	Source/composition	Behavioral evaluations	Main mechanism of action	Reference
Resveratrol	Tiger Balm, Cassia and many other plants	OFT, EPM, TST, FST	Modulation of SIRT1/NF-κB signaling pathway to inhibit inflammatory response	[98]
		RIT, LDT, OFT, SPT, FST	Improved brain antioxidant and monoamine levels and HPA axis	[99]
Fenugreek chloride	Fenugreek	FST, OFT, Splash Test, EPM	Relieves oxidative stress and increases antioxidant capacity	[103]
Ganoderma triterpenes (botany)	Commonly found in the plant kingdom and most fruits and vegetables	OFT, EPM, Splash Test, SPT, FST, TST, Nesting Test	Inhibition of inflammatory response and microglia activation	[106]
Oleanolic acid and ursolic acid	Commonly found in the plant kingdom and in most fruits and vegetables	OFT, EPM, FST, Splash Test	Suppresses neuroinflammation and improves synaptic plasticity	[109]
Paeonia lactiflora total	Root of herbaceous peony (Paeonia lactiflora), used in TCM	OFT, Social Interaction Experiment	Inhibition of microglial	[111]
			Activation of cellular functions	
Soy isoflavone	Soybeans	FST, TST	Increased serum dopamine and estrogen receptor beta levels	[113]
Four inverted powder (TCM)	Bupleurum, peony	OFT, EPM, SPT, TST, FST	Regulation of Rac1 activity and dendritic spine plasticity by NAc	[115]
	citrus fruit, licorice	SPT, OFT, FST	Regulation of BDNF/PKA/CREB signaling pathway	[116]
		SPT, OFT, FST	Activation of CaSR/PKC/ERK signaling pathway improves synaptic plasticity	[117]
		SPT, OFT, FST	Regulation of mitochondrial function and synaptic plasticity	[118]
Warming Yang and Relieving Depression Formula	Angelica	OFT	Regulation of the HPA axis, modulation of neuroplasticity	[118]
	Radix	OFT, O Maze Experiment, Social Interaction Cognitive Experiment	Inhibition of hippocampal microglia activation and alleviation of overactive neuroinflammation	[122]
	Bai Shao			
	Rhizoma			
	Poria			
	Ginger			
	Mentha			
	Radix Glycyrrhiza			
	Radix Bupleurum			
	Radix Cynomorium			
	Herba Epimedium			
	Radix Morinda			
Kaixin San	Ginseng, Acorus calamus, Poria cocos and Polygalactus	FST, OFT	Regulate lipid balance	[123]
		FST, SPT		[125]

OFT, Open Field Test; EPM, Elevated Plus Maze; TST, Tail Suspension Test; FST, Forced Swim Test; LDT, Light/Dark Box Test; RIT, Resident Intruder Test; SPT, Sucrose Preference Test; FST, Forced Swim Test; SIRT1, sirtuin 1; BDNF, brain-derived neurotrophic Factor; PKA, protein kinase A; CREB, cAMP response element-binding protein; HPA, hypothalamic-pituitary-adrenal Axis; NAc, nucleus accumbens; CaSR, calcium-sensing receptor; ERK, extracellular signal-regulated kinase; PKC, protein kinase C.

## 4. Discussion

Depression is one of the most common mental disorders and eventually leads to suicidal thoughts or behavior [126]. The prevention and treatment of depression is a constant challenge for modern medicine, as there is currently no treatment that successfully prevents or completely reverses depression and the pathogenesis of depression is still opaque. Early life experiences have a significant impact on the neurological, behavioral, and psychological development of children and have a lasting effect in many areas [127]. MS is a commonly used modeling method in ELS and involved in the pathogenesis and development of depression by mediating neuroinflammation, neuroplasticity, the HPA axis, neurotransmitters, the microbe-gut-brain axis, epigenetics, autophagy, and circadian rhythms. TCM has significant clinical efficacy and has the advantage of multiple targets, multiple links, and multiple levels. The use of TCM in anti-depression research, scientifically explaining the treatment rules of TCM and providing an alternative for Western medicine’s single component and single target approach, should be the focus of current research. Studies have shown that in the MS-induced depression model, TCM compounds and active ingredients have antidepressant effects.

However, currently, most of the research on MS-induced depression focuses on preclinical studies and there is a lack of relevant high-quality studies at the clinical level. Further, self-reports and clinical interviews are common means to assess ELS. Additionally, the assessment of ELS involves multidisciplinary methods such as psychology, neuroscience, and physiology. However, there are neither current standards nor recognized assessment methods for the evaluation of the ELS experience of patients with depression.

In preclinical studies, the majority of investigators only explore the mechanism of action of TCM, while the relationship between TCM theory, MS, and depression has not been explored. Furthermore, some TCMs lack quality control (specifically manifested as an absence of unified and standardized methods for the cultivation and harvesting of drugs, resulting in batch differences in active ingredients). The inconsistent processing techniques of TCM affect its stability and efficacy. The lack of standardized detection methods for fingerprint spectra cannot guarantee the consistency and stability of chemical components, making the study of its mechanism of action difficult.

Meanwhile, whether some active ingredients of TCM target specific locations within the central nervous system remains to be clarified. More importantly, MS-induced depression involves a complex process involving multiple signaling pathways and coordination among cells. However, the majority of current research on TCM focuses on single signaling pathways, with limited detection indicators. This fails to fully reveal the characteristics of the multi-target and multi-pathway effects of TCM, which limits both the breadth and depth of research.

High-quality clinical trials should be undertaken with the guidance of TCM theory to further reveal the role of TCM in patients with depression. Additionally, research on targeted drug delivery systems for TCM should be strengthened and the quality control standards for Chinese medicinal materials improved (for example, promoting the certification of standardized planting bases; formulation of standardized processing technology; establishment of a quality evaluation system based on fingerprint spectra). Meanwhile, technologies such as single-cell sequencing and spatial transcriptomics should be combined to comprehensively reveal the potential molecular mechanism of TCM in treating MS depression and to improve the biological implications of the TCM theory behind it. In conclusion, the relationship between MS and depression as well as the mechanism of action of TCM still require considerable research.

## 5. Conclusion

Maternal separation mediates the occurrence of depression through dysregulation across multiple pathways, including neuroinflammation, impaired synaptic plasticity, hyperactivity of the HPA axis, neurotransmitter dysfunction, gut-brain axis dysregulation, epigenetic modifications, autophagic disorders, and circadian rhythm imbalance. Preclinical studies have confirmed that traditional Chinese medicine monomers (e.g., resveratrol and Ganoderma lucidum triterpenoids) and compound prescriptions (e.g., Sini San and Kaixin San) possess multi-target therapeutic potential. However, their clinical translation is hindered by fluctuations in medicinal material quality (attributable to insufficient standardization in cultivation and processing) and a lack of robust clinical efficacy evidence. Future research should focus on overcoming these limitations to advance the development of precise diagnostics and therapeutics for depression.
